# A Transdiagnostic Self-management Web-Based App for Sleep Disturbance in Adolescents and Young Adults: Feasibility and Acceptability Study

**DOI:** 10.2196/25392

**Published:** 2021-11-01

**Authors:** Nicole E Carmona, Aleksandra Usyatynsky, Samlau Kutana, Penny Corkum, Joanna Henderson, Kelly McShane, Colin Shapiro, Souraya Sidani, Jennifer Stinson, Colleen E Carney

**Affiliations:** 1 Department of Psychology Ryerson University Toronto, ON Canada; 2 Department of Psychology Memorial University of Newfoundland St. John's, NL Canada; 3 Department of Psychology and Neuroscience Dalhousie University Halifax, NS Canada; 4 Department of Psychiatry Dalhousie University Halifax, NS Canada; 5 Department of Psychiatry University of Toronto Toronto, ON Canada; 6 Margaret and Wallace McCain Centre for Child, Youth and Family Mental Health Centre for Addiction and Mental Health Toronto, ON Canada; 7 Human Resource Management and Organizational Behaviour Ted Rogers School of Management Ryerson University Toronto, ON Canada; 8 Department of Ophthamology University of Toronto Toronto, ON Canada; 9 Daphne Cockwell School of Nursing Ryerson University Toronto, ON Canada; 10 Chronic Pain Program Department of Anesthesia and Pain Medicine The Hospital for Sick Children Toronto, ON Canada; 11 Child Health Evaluative Sciences Research Institute The Hospital for Sick Children Toronto, ON Canada; 12 Lawrence S Bloomberg Faculty of Nursing University of Toronto Toronto, ON Canada

**Keywords:** youth, sleep, technology, mHealth, self-management, adolescents, young adults, mobile phone

## Abstract

**Background:**

Sleep disturbance and its daytime sequelae, which comprise complex, transdiagnostic sleep problems, are pervasive problems in adolescents and young adults (AYAs) and are associated with negative outcomes. Effective interventions must be both evidence based and individually tailored. Some AYAs prefer self-management and digital approaches. Leveraging these preferences is helpful, given the dearth of AYA treatment providers trained in behavioral sleep medicine. We involved AYAs in the co-design of a behavioral, self-management, transdiagnostic sleep app called DOZE (Delivering Online Zzz’s with Empirical Support).

**Objective:**

This study tests the feasibility and acceptability of DOZE in a community AYA sample aged 15-24 years. The secondary objective is to evaluate sleep and related outcomes in this nonclinical sample.

**Methods:**

Participants used DOZE for 4 weeks (2 periods of 2 weeks). They completed sleep diaries, received feedback on their sleep, set goals in identified target areas, and accessed tips to help them achieve their goals. Measures of acceptability and credibility were completed at baseline and end point. Google Analytics was used to understand the patterns of app use to assess feasibility. Participants completed questionnaires assessing fatigue, sleepiness, chronotype, depression, anxiety, and quality of life at baseline and end point.

**Results:**

In total, 83 participants created a DOZE account, and 51 completed the study. During the study, 2659 app sessions took place with an average duration of 3:02 minutes. AYAs tracked most days in period 1 (mean 10.52, SD 4.87) and period 2 (mean 9.81, SD 6.65), with a modal time of 9 AM (within 2 hours of waking). DOZE was appraised as highly acceptable (mode≥4) on the items “easy to use,” “easy to understand,” “time commitment,” and “overall satisfaction” and was rated as credible (mode≥4) at baseline and end point across all items (logic, confident it would work, confident recommending it to a friend, willingness to undergo, and perceived success in treating others). The most common goals set were decreasing schedule variability (34/83, 41% of participants), naps (17/83, 20%), and morning lingering in bed (16/83, 19%). AYAs accessed tips on difficulty winding down (24/83, 29% of participants), being a night owl (17/83, 20%), difficulty getting up (13/83, 16%), and fatigue (13/83, 16%). There were significant improvements in morning lingering in bed (*P=*.03); total wake time (*P=*.02); sleep efficiency (*P=*.002); total sleep time (*P*=.03); and self-reported insomnia severity (*P=*.001), anxiety (*P=*.002), depression (*P=*.004), and energy (*P=*.01).

**Conclusions:**

Our results support the feasibility, acceptability, credibility, and preliminary efficacy of DOZE. AYAs are able to set and achieve goals based on tailored feedback on their sleep habits, which is consistent with research suggesting that AYAs prefer autonomy in their health care choices and produce good results when given tools that support their autonomy.

**Trial Registration:**

ClinicalTrials.gov NCT03960294; https://clinicaltrials.gov/ct2/show/NCT03960294

## Introduction

### Background

Emerging adulthood is a time of risk for sleep problems. An epidemic of shortened sleep times and resultant sleepiness in teens and young adults has been reported worldwide [1,2]. Sleep problems are reported in two-thirds of adolescents and young adults (AYAs) [3-5] and are linked to academic and mental health problems and suicidality [6-11]. AYAs also experience a pubertal shift toward later or delayed circadian rhythmicity, such that they become tired later, resulting in later rise times, although they must maintain early rise times to attend school [12-15]. This biological shift toward a later sleep onset time is further compounded by the fact that AYAs are sensitive to evening light [12,16,17] and are exposed to academic (eg, early school start times) and social pressures (eg, peers reinforcing later activities), which can increase sleeplessness. Moreover, some AYAs exert control over what they eat and drink (eg, caffeine) without an adequate understanding of the consequences of particular behaviors on their sleep and general health [18]. As a result, AYAs have chronic sleep loss, resulting in pathological sleepiness, a condition associated with an increased risk of motor vehicle accidents and poor emotional, interpersonal, and academic functioning [19,20].

Providing accessible interventions for sleep problems is crucial to mitigate personal, social, and economic costs. Treatment options for most sleep problems typically consist of pharmacological and behavioral approaches [21,22]. Sleep medications are associated with significant concerns regarding use by AYAs [23]. Although cognitive behavioral therapy for insomnia (CBT-I) approaches are the most efficacious for helping individuals with insomnia with few troublesome side effects [24], few AYAs have access to behavioral sleep medicine treatments [25,26]. Furthermore, CBT-I protocols (when accessible) do not target the range of sleep problems that AYAs experience beyond insomnia symptoms, including hypersomnia, excessive daytime sleepiness (eg, from voluntary sleep restriction), delayed phase circadian rhythm, and often uninformed use of sleep-interfering substances, some of which may be experienced concurrently, highlighting the promise of transdiagnostic behavioral sleep treatments for AYAs [27-29]. There are multiple barriers to accessing nonpharmacological evidence-based sleep treatments, including poor dissemination of CBT-I and behavioral sleep treatments in real-world settings [30] and a shortage of providers who are trained in such treatments [31]. It is clear that AYAs have diverse sleep problems (ie, not just insomnia) and require tailored evidence-based strategies that comprehensively address these diverse needs, making transdiagnostic cognitive behavioral treatments [32] a more suitable option than nonadapted CBT-I.

Smartphone apps are uniquely suited to improve access for those who are not seeking treatment but are nevertheless at risk as well as treatment-seeking groups with poor access to evidence-based treatments, and reportedly increase users’ motivation, goal-setting, and confidence in and control over their ability to enact health-promoting behavior change [33]. AYAs are drawn to technology; therefore, technology provides an opportunity to directly reach this large invisible at-risk cohort for whom sleep disorders contribute to psychopathology and vice versa [34,35]. Although there are web-based programs for infant (aged 6-36 months) [36], pediatric (aged 1-8 years) [37,38], adolescent [39-41], and adult sleep disturbances [42], there are no evidence-based transdiagnostic programs that adequately address the *multitude* of sleep problems observed in AYAs and that span both adolescence and young adulthood. Put differently, as some of the sleep problems of AYAs are unique and go beyond insomnia (eg, voluntary sleep restriction, circadian phase delay, and poor sleep hygiene), pediatric and adult programs are not suitable for this age group.

### Objective

In response to a dearth of appropriate treatment options for AYAs with a range of sleep difficulties beyond insomnia, we created a self-management app called DOZE (Delivering Online Zzz’s with Empirical Support), which was co-designed by AYAs in user consultation sessions conducted by the developers of the app. DOZE is based on the principles of cognitive behavioral sleep medicine [32] and allows AYAs access to effective, tailored strategies to address a range of sleep problems (eg, insomnia, daytime sleepiness, and delayed phase circadian rhythms), which also range from subclinical to clinical in terms of their severity. Importantly, consistent with their preferences for technology and autonomy and as revealed by the user consultation sessions, the self-management approach entrusts AYAs to set their own goals and make their own health behavior changes when provided with the tools and education to do so. This study reports on an open trial evaluating the app’s feasibility over a 4-week intervention period. The primary objective of this study is to evaluate whether AYAs regard evidence-based cognitive behavioral strategies in a self-management app (DOZE) as acceptable and credible. Toward the aim of evaluating feasibility, we also want to understand how AYAs use DOZE and which aspects of the app they find most valuable. As AYAs co-designed the app, we hypothesize that AYAs would rate the app as acceptable and credible and report high satisfaction. No a priori hypotheses are made regarding which aspects of the app would be most valuable to AYAs, and an exploratory analysis of app use patterns is conducted to understand which aspects of the app are most useful and to provide further insight into AYAs’ primary sleep complaints and treatment goals.

The secondary objective is to examine preliminary efficacy, acknowledging that these results may be quite modest because of the range of sleep problems addressed (eg, both insomnia and hypersomnia) and that, without sleep disturbance severity as part of the inclusion criteria, we might recruit a subclinical sample. Outcomes focus on whether DOZE positively affects health-related behavior change, improvements in daytime symptoms (ie, sleepiness, fatigue, energy, and psychological symptoms), and health-related quality of life. We hypothesize that AYAs who use DOZE would see improvements in sleep indices at end point. Furthermore, we hypothesize that the use of DOZE for 4 weeks may contribute to improvements in energy, mood, and health-related quality of life at end point compared with baseline.

## Methods

### Participants

Individuals between the ages of 15 and 24 years with self-reported dissatisfaction with their sleep were eligible to participate in this web-based study. Given that a clinical diagnosis of a sleep disorder is not required to use DOZE (ie, any AYA who wants to improve sleep can use it), this study did not have any other inclusion or exclusion criteria to be able to generalize the results of this study to the population that will use DOZE. Self-reported dissatisfaction with sleep was queried with a single question, “How do you feel about your sleep?” and participants could respond using an open textbox. Computer and internet literacy of participants was assumed. Participants were recruited using posters at Ryerson University and in the downtown Toronto community, dissemination of study information to mental health providers via listservs of professional organizations (eg, Canadian Association for Cognitive and Behavioural Therapy and Ontario Psychological Association), and via social media (eg, Twitter and Facebook). Recruitment materials advertised the opportunity for AYAs who were dissatisfied with their sleep or were experiencing difficulty sleeping to participate in a study testing a new sleep app for 4 weeks. Participation in this study was quasi-anonymous. Email confirmation was used to detect repeat participation. All participants provided informed consent and completed a quiz assessing their understanding of critical components of the consent form (detailed in the *Procedure* section).

### Intervention: DOZE App

DOZE [43] is a transdiagnostic, self-management, cognitive behavioral web-based app for sleep disturbance among AYAs. It is freely accessed from a web browser on any device (ie, it is not downloaded on a device). DOZE was designed using an iterative, experience-based co-design process that included feedback from AYA users and health care provider stakeholders [44-46]. Initial design consultation interviews conducted by an industry partner with AYA stakeholders revealed that, in addition to including a sleep diary for self-monitoring, AYAs wanted an app that provides feedback on their personal sleep habits (over and above feedback on their sleep), opportunities for goal-setting, and tips on how to make changes to their sleep. Following this initial phase, a user-informed redesign of DOZE was conducted with an industry partner (PIVOT Design), consistent with standard app development processes [47], including 6 user assessments with AYAs (aged 15-24 years) on iterative prototype versions of the app and consultations with health care provider stakeholders. Of note, AYAs who contributed to the co-design of DOZE were not participants in this study. Additional details about the co-design process are presented in Multimedia Appendix 1.

The final version of DOZE includes a sleep diary, adapted with permission from the Consensus Sleep Diary [48], comprising 8 questions that assess the following: bedtime (ie, time of getting into bed whether or not it was to initiate sleep), time of sleep attempt (ie, time of trying to sleep), time it took to fall asleep (sleep onset latency [SOL]), total time awake in the night (wake after sleep onset), time of final awakening (ie, time of waking in the morning), rise time (ie, time of getting out of bed), amount of time spent napping, and use of sleep-interfering substances within 2 hours of bedtime. DOZE’s onboarding screens instructed participants to complete their sleep diary every morning within 2 hours of waking, and they were reminded of this using a pop-up notification if they completed it that same day outside of the 2-hour window. Completing an entry for the previous day was not permitted. Participants also received an automated reminder email each morning to complete the diary. Sleep indices were calculated based on information provided by the sleep diary, including SOL, variability in rise time (range of earliest to latest rise time), variability in bedtime (range of earliest to latest bedtime), wake after sleep onset, morning lingering in bed (the difference between time of final awakening and rise time), total sleep time (TST; total time spent asleep), total wake time (TWT; total time spent awake during the sleep period), time in bed (TIB; bedtime to rise time), and sleep efficiency (SE; percent of TIB spent asleep). Participants were able to view their average sleep indices over a 2-week period on a personalized dashboard.

**Figure 1:**
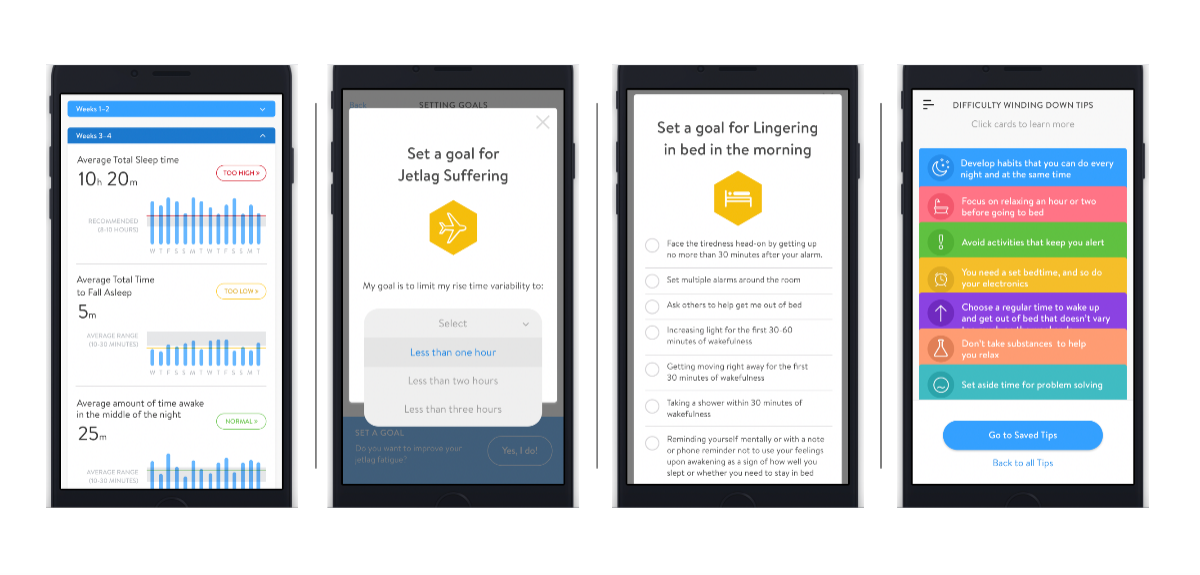
Screenshots of Delivering Online Zzz’s with Empirical Support: progress and feedback, goal-setting, and tips.

After completing 2 weeks of sleep diaries, DOZE provides personalized feedback on sleep indices (TST, SOL, TWT, TIB, and SE) relative to age-adjusted norms [4] by indicating whether their indices are normal, too high, or too low on the Progress screen. Users then have the opportunity to set goals to improve their sleep in *problem areas* that are identified (ie, indices are outside the normal range). Feedback areas, which all represent components of evidence-based treatment [32], include *naps*, *too much/too little TIB*, *sleep-interfering substances*, *lingering in bed in the morning*, *sleepiness*, and *jetlag without traveling* (ie, sleep schedule variability). AYAs have the opportunity to set personalized goals in these areas (eg, reduce jetlag by 1 hour, 2 hours, or 3 hours), which are added to their personal dashboard, or they may choose not to set goals in identified areas, accounting for various stages of readiness to change. Notably, if a feedback area is not relevant to the individual based on their sleep indices (eg, they are spending a normal amount of TIB), they are not provided the opportunity to set a goal to improve in that area (eg, they are not shown the feedback area *too much/too little TIB*). In addition, participants could access a Tips section that included quizzes and psychoeducation about hyperarousal (*difficulty winding down*), chronotype (*night owl living in an early bird’s world*), difficulty getting out of bed in the morning, daytime sleepiness (*trouble staying awake during the day*), and fatigue (*exhausted*). Tips can also be saved to participants’ dashboards for a personalized user experience, and all participants have access to all of the tips. After setting goals, participants then complete sleep diaries for an additional 2 weeks and, at the conclusion of the second 2-week period, they are able to see a comparison of their progress between weeks 1-2 and weeks 3-4 on their dashboard. Screenshots of the example progress, goal-setting, and tips screens are presented in Figure 1. Participants did not receive any other support or interventions in this study.

### Measures

Outcomes were assessed using analysis of aggregate DOZE use during the study period and through the completion of web-based qualitative and quantitative questionnaires.

#### Feasibility and Acceptability

Treatment acceptability, credibility, and satisfaction were assessed using both quantitative and qualitative measures. A 5-item Treatment Evaluation Questionnaire (TEQ) was administered at baseline and end point and assessed participants’ perceptions of DOZE as a logical treatment, beliefs that it would be successful, confidence in referring it to a friend, willingness to undergo treatment, and whether they thought it was successful in treating others [49]. As reviewed in the *Introduction* section, DOZE is a self-management intervention rather than a treatment for clinical sleep disorders. The use of the word *treatment* in evaluating the perceived credibility of DOZE reflects the verbatim wording of each item of the TEQ, which has been used in previous clinical trials of sleep medicine treatments [50]. Each item is scored from 1 (*not at all*) to 7 (*very*), with higher scores reflecting greater perceived credibility and satisfaction with DOZE. In addition, a 6-item Acceptability Scale was designed for the purpose of this study and administered at end point. This measure assessed participants’ attitudes regarding ease of use, understandability, enjoyment, perceived helpfulness, time commitment, and overall satisfaction. Participants rated each item on a scale of 1-5, with higher scores denoting greater acceptability and more favorable attitudes toward DOZE. In addition, participants completed a qualitative exit questionnaire about their experience using DOZE at end point, including how they felt about their sleep and the goals they set, what they liked and did not like, and whether there was anything they would change about DOZE. An analysis of qualitative responses will be reported in a separate paper.

Google Analytics was used to provide additional information about DOZE use, including the number of page views; where, when, and how DOZE was accessed; and the mean length of sessions using DOZE. Consistent with what is available on the Google Analytics website, only averages are provided, as other measures of central tendency are unavailable.

#### Preliminary Efficacy

Improvements in sleep and sleep-related behaviors were evaluated by analyzing changes in the following sleep and behavioral indices derived from the sleep diary: variability in bedtime, variability in sleep attempt, variability in wake time, variability in rise time, morning lingering in bed, SE, TST, TWT, TIB, duration of naps, and frequency of sleep-interfering substance use. In addition, participants completed self-report questionnaires at baseline and end point to investigate whether the use of DOZE for 4 weeks contributed to improvements in insomnia severity, chronotype, daytime symptoms, and health-related quality of life.

The Insomnia Severity Index (ISI [51]) is a 7-item scale that evaluates self-reported sleep disturbances. Items are scored on a 5-point Likert scale from 0 (*no sleep difficulty*) to 4 (*severe sleep difficulty*), with total scores ranging from 0 to 28. A cutoff score of 10 distinguishes between good and poor sleepers in adult community samples [52]. Previous research has suggested a cutoff score of 9 among adolescent samples [53]; however, because of the larger proportion of young adults in our sample relative to adolescents (see the *User Characteristics* section), we used the more conservative cutoff score of 10 with the understanding that clinical levels of insomnia may be underestimated in our sample. The ISI has been validated in AYA samples and is a validated outcome measure with good psychometric properties [53-56].

Chronotype, which refers to one’s endogenous circadian preference, was measured using the 13-item Composite Scale of Morningness (CSM [57]). Total scores range from 13 to 55, with higher scores reflecting greater morningness preference; based on established cutoffs, scores between 44 and 55 indicate morning type, between 23 and 43 indicate intermediate type, and between 13 and 22 reflect evening type [57]. The CSM has been validated in samples of adolescents and undergraduate students [57-59].

Fatigue was measured using the Fatigue Severity Scale (FSS [60]). The FSS is a 9-item scale measuring impairment related to fatigue, and items are rated on a Likert scale from 1 (*no impairment*) to 7 (*severe impairment*). A mean item score is calculated, with higher scores indicating more severe fatigue. The FSS has demonstrated convergent validity with a visual analog scale for fatigue [60] and has previously been used to measure fatigue in clinical samples of adolescents [61,62].

Daytime sleepiness was measured using the Cleveland Adolescent Sleepiness Questionnaire (CASQ) [63], which comprises 16 items scored on a 5-point Likert scale ranging from *never* to *almost every day*. Items are scored to yield a total score, as well as a Sleepiness subscale and a reverse-coded Alertness subscale, with higher scores indicating greater daytime sleepiness. The CASQ has been validated in samples of clinical and nonclinical adolescents [63].

The Center for Epidemiologic Studies Depression Scale–Revised 10-item Version for Adolescents (CESDR-10 [64]) was included to evaluate depression symptom severity. The item assessing suicidality was removed to mitigate risk (because of the nature of the web-based study), leaving a 9-item scale. Participants rated the frequency of each symptom during the past 2 weeks, ranging from 0 (*not at all or less than 1 day*) to 4 (*nearly every day for 2 weeks*). In this study, total scores ranged from 0 to 36, with higher scores reflecting greater depression symptom severity. The CESDR-10 has demonstrated excellent internal consistency in adolescents and is a valid screening measure for depression [64].

Anxiety symptoms were assessed using the State-Trait Inventory of Cognitive and Somatic Anxiety (STICSA [65]), which comprises scales assessing the presence of cognitive and somatic symptoms of anxiety at the moment it is being completed (State) and in general (Trait). The State and Trait scales include 21 statements, and agreement is rated on a scale from 1 (*not at all*) to 4 (*very much so*). Total scores on both scales range from 21 to 84. The STICSA has demonstrated reliability and validity in undergraduate and adolescent samples [66-68] and has demonstrated sensitivity to change [68].

The RAND 36-item Short Form Health Survey 1.0 (SF-36 [69]) was included in this study to evaluate health-related quality of life. The SF-36 consists of 36 items that assess the following domains (scales): physical functioning, role functioning–physical, role functioning-emotional, energy, emotional well-being, social functioning, pain, and general health. Each scale score ranges from 0-110, with higher scores denoting better health functioning. Each scale has demonstrated adequate to excellent internal consistency [70].

### Procedure

Interested AYAs were directed to the DOZE website, where they were provided with a consent form and details of our privacy policy. As the study took place on the web, a brief quiz followed the consent form to ensure that participants understood key aspects of informed consent, including the purpose of the study, risks and benefits of participation, and limits of confidentiality. After the provision of informed consent, participants received a randomly generated 5-digit study ID and completed a battery of questionnaires using Qualtrics, which included a demographic form and the ISI, FSS, CSM, CASQ, CESDR-10, STICSA State and Trait, SF-36, and TEQ. At the end of the questionnaires, participants received a link to access DOZE and create an account.

As indicated by the in-app onboarding process, participants were instructed to use DOZE for 2 weeks (ie, period 1), after which they would receive personalized feedback on their sleep, have the opportunity to set goals and access tips, and track their sleep for 2 more weeks (ie, period 2) to examine their progress. Participants were contacted via email by the study coordinator to maintain adherence with sleep tracking if they missed several consecutive days of completing the sleep diary and were sent a standardized email encouraging them to get back on track the next day. Following the completion of the 4-week study period, participants were emailed a link to complete the web-based posttest battery of questionnaires that included the ISI, FSS, CSM, CASQ, CESDR-10, STICSA State and Trait, SF-36, and TEQ, as well as the Acceptability Scale and the Qualitative Exit Questionnaire. Participants were compensated CAD $12.50 (US $9.79) for each study component that they completed; thus, participants who completed all 4 components of the study were emailed a CAD $50 (US $39.17) gift card honorarium for their participation. This study was approved by the research ethics board of Ryerson University, Toronto, Canada. Data collection took place between September 2019 and January 2020. There were no significant changes to the methods of the study or the intervention (eg, bug fixes, downtimes, or content changes) while this study was underway.

### Statistical Analysis

User characteristics, including self-reported demographic and baseline clinical characteristics as well as sleep indices from period 1, were evaluated using descriptive statistics for all participants who created a DOZE account. The demographic characteristics and scores on all self-report measures at baseline were compared between early dropouts (ie, before, during, and after the baseline questionnaires) and those who went on to create a DOZE account to assess for possible differences, as well as between all dropouts and those who completed the study (*study finishers*). To evaluate the feasibility of DOZE, including the characteristics and app use patterns of all DOZE users, analyses of app use (ie, number of days of sleep diary completion, use of goals and tips, and Google Analytics) included participants who created an app account regardless of whether or not they completed the study. Histograms and P-P plots were generated for visual inspection of normality, and extreme outliers on sleep indices were removed on the basis of visual inspection of boxplots. Descriptive statistics were used to analyze demographic and clinical characteristics. Patterns of app use were analyzed using descriptive statistics and Google Analytics.

Owing to insufficient sleep diary data from DOZE users who did not complete the study (32/83, 39%), sleep diary indices are only presented for study finishers. In addition, analyses evaluating acceptability, credibility, and satisfaction with DOZE, and examining the efficacy of DOZE in improving sleep indices and self-reported measures of sleep and daytime functioning were only conducted among study finishers. Paired sample 2-tailed *t* tests were used to analyze changes in satisfaction with DOZE from baseline to end point, and acceptability was analyzed using descriptive statistics. Changes in sleep indices from period 1 to period 2 were analyzed using paired sample *t* tests. Similarly, paired sample *t* tests with 1000 bootstrapped samples were used to evaluate differences in self-reported insomnia severity, daytime symptoms, and health-related quality of life from baseline to end point to account for slight deviations from normality. Effect sizes were calculated for all analyses of change from baseline to end point using Cohen *d* for repeated measures. Unless otherwise specified, all analyses were performed using SPSS for Mac version 26 (IBM Corporation).

## Results

### User Characteristics

**Figure 2:**
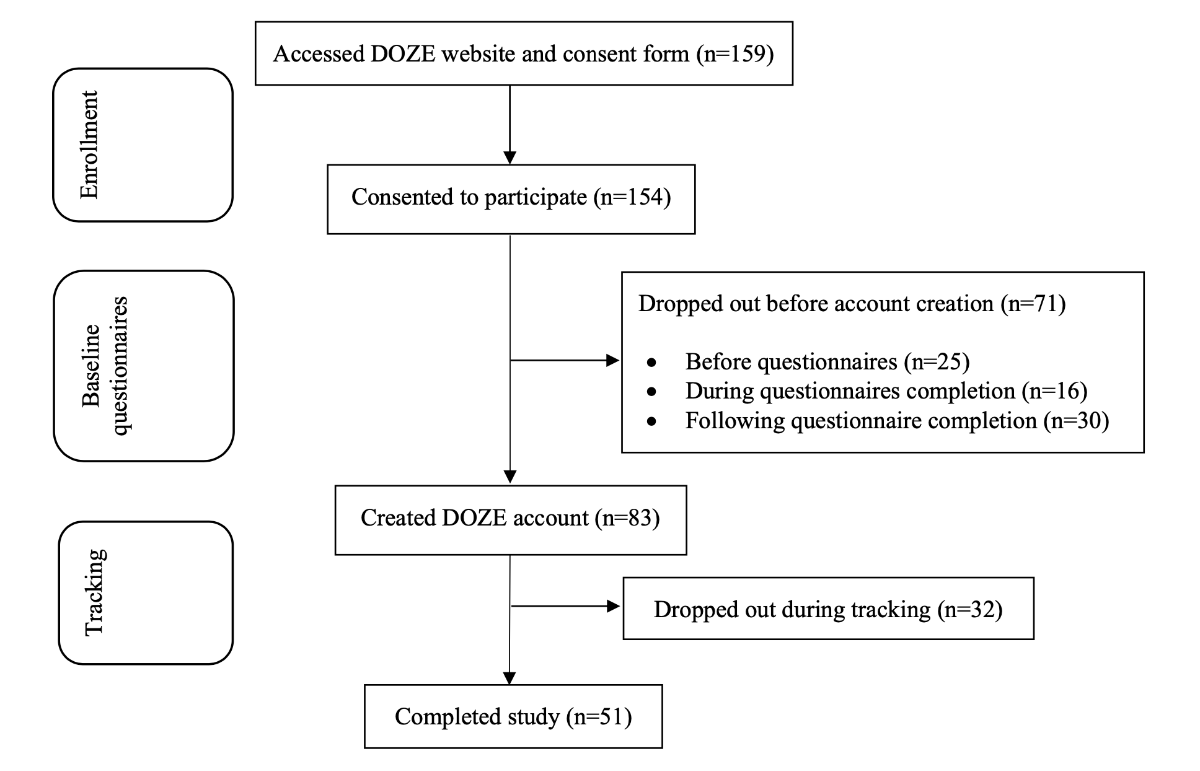
Flowchart of participants. DOZE: Delivering Online Zzz’s with Empirical Support.

In total, 154 participants consented to participate in this study (Figure 2). Of the 154 participants who consented to participate, 71 (46.1%) dropped out during the baseline questionnaire process. Over half of the participants (83/154, 53.9%) created an app account and completed some tracking. Approximately 33.1% (51/154) of participants completed all 4 components of the study (baseline questionnaires, first 2 weeks of sleep diaries, second 2 weeks of sleep diaries, and end point questionnaires).

Demographic characteristics were analyzed using data from all DOZE users (83/83, 100%; Table 1). Participants were on average 20.17 years (SD 2.49 years). Out of the 83 participants, 20 (24%) were adolescents (between the ages of 15 and 18 years) and 63 (76%) were young adults (between the ages of 19 and 24 years). The sample was predominantly female (57/83, 69%) and European Canadian (37/83, 45%). Most participants were not currently seeing a health care professional for the purpose of a sleep problem (79/83, 95%) or a mental disorder (69/83, 83%). Despite low levels of treatment-seeking, the average score on the ISI indicated sleep disturbance in the mild range (mean 12.48, SD 4.67), and 76% (63/83) of participants exceeded the clinical cutoff on the ISI (ie, score>10). In addition, the sample was characterized by high levels of fatigue, as indicated by the FSS (mean 4.73, SD 1.01) and the energy scale of the SF-36 (mean 35.71, SD 18.24) and daytime sleepiness (mean 45.14, SD 9.47) and on average had an intermediate chronotype on the CSM (mean 30.07, SD 7.16). Levels of state (mean 42.25, SD 12.73) and trait anxiety (mean 42.71, SD 13.15), as well as depression (mean 16.85, SD 8.30), were mild in this sample. Notably, early dropouts of the study (ie, before, during, and after the baseline questionnaires) did not differ from DOZE users on age (t_114_=0.40; *P=*.69), gender (*χ*^2^_2_=3.0; *P=*.22), ethnicity (*χ*^2^_9_=16.3; *P=*.06), ISI (t_124_=1.17; *P=*.25), FSS (t_124_=1.08; *P*=.28), CMQ (t_118_=0.63; *P*=.53), CASQ (t_113_=−0.60; *P*=.55), STICSA State (t_111_=0.74; *P*=.46), STICSA Trait (t_104_=0.41; *P*=.69), CESDR-10 (t_115_=−0.03; *P*=.98), or any of the SF-36 scales (*P* values range from .55 to .99). Similarly, study finishers did not differ from participants who dropped out at any point on any of these demographic or clinical characteristics (data not shown).

**Table 1:** Demographic characteristics of participants at baseline (N=83).

Baseline characteristics		Participants, n (%)
**Sex**		
	Female	57 (69)
	Male	26 (31)
**Ethnicity**		
	Caribbean Canadian	1 (1)
	East or Southeast Asian Canadian	15 (18)
	European Canadian	37 (45)
	Latin American, Central American, or South American Canadian	4 (5)
	South Asian Canadian	8 (10)
	West Asian or Arab Canadian	1 (1)
	Pacific Islander Canadian	1 (1)
	Other	16 (19)
Seeing an HCP^a^ for sleep disorder^b^		4 (5)
Seeing an HCP for mental disorder^b^		14 (17)

^a^HCP: health care professional.

^b^Reflects the number and percentage of participants who answered "yes" when asked about whether they were currently seeing a health care professional for this issue.

We analyzed sleep indices for study finishers at periods 1 and 2 (Table 2). After removing outliers (ranging from n=1 to n=6 depending on the sleep index), period 1 sleep indices indicated that despite an average SE in the normal range (ie, >85%), DOZE users spent a clinically meaningful amount of time awake at night. DOZE users also had variable sleep schedules, characterized by >4 hours of variability in their bedtime, sleep attempt, wake time, and rise time over a 2-week period. In addition, DOZE users obtained 7 hours 20 minutes of sleep on average despite spending 8 hours 21 minutes in bed; a one-sample *t* test indicated that this is significantly lower than 8.5, the minimum recommended amount of TST for AYAs (t_50_=−8.76; *P*<.001). In addition, DOZE users spent an average of nearly half an hour lingering in bed in the morning.

**Table 2:** Change in sleep indices for DOZE (Delivering Online Zzz’s with Empirical Support) study finishers^a^.

Sleep diary variable	Period 1, mean (SD)	Period 2, mean (SD)	*t* test^b^ (*df*)	*P* value	Cohen *d*
Bedtime variability (hours)	4.26 (2.16)	4.96 (4.08)	0.52 (49)	.60	0.07
Sleep attempt variability (hours)	4.04 (2.19)	4.01 (2.13)	0.88 (49)	.38	0.12
Wake time variability (hours)	4.59 (2.47)	4.62 (2.38)	−0.22 (49)	.83	0.03
Rise time variability (hours)	4.71 (2.41)	5.05 (2.83)	−0.13 (49)	.90	0.02
Nap duration (hours)	0.370 (0.50)	0.220 (0.32)	1.63 (32)	.11	0.28
Morning lingering in bed (hours)	0.402 (0.28)	0.353 (0.30)	2.24 (46)	.03	0.30
Time in bed (hours)	8.35 (1.06)	8.49 (0.85)	−2.40 (49)	.02	0.34
Total sleep time (hours)	7.34 (0.93)	7.67 (0.97)	−4.23 (49)	<.001	0.60
Total wake time (hours)	1.00 (0.54)	0.837 (0.55)	2.54 (47)	.01	0.36
Sleep efficiency (%)	87.67 (6.44)	90.18 (6.46)	−3.43 (49)	.001	0.48
Frequency of sleep-interfering substance use^c^	0.142 (0.21)	0.131 (0.21)	0.34 (49)^d^	.74	0.05

^a^Test statistic and Cohen *d* are for log-transformed variables, whereas means and SDs refer to untransformed variables.

^b^Test is 2-tailed.

^c^Frequency of substance use refers to the proportion of days within a period in which sleep-interfering substance use was endorsed in the sleep diary.

^d^Not log-transformed.

### Feasibility and Acceptability

#### Feasibility

Among all participants who created an app account, sleep diaries were completed for an average of 10.52 days (SD 4.87 days; range 0-14 days; 82/83, 99%) in period 1 and 9.81 days (SD 6.65 days; range 0-2 days; 75/83, 90%) in period 2. Over two-third of app users (57/83, 69%) used DOZE long enough to receive feedback on their sleep and to set goals; of these 57 DOZE users, 46 (81%) chose to set one or more goals to improve their sleep. Participants set an average of 1.54 goals (SD 1.12; range 0-4 goals; 57/83, 69%) following period 1.

The most frequent goal was to reduce schedule variability (jetlag), followed by reducing naps and reducing lingering in bed in the morning (Table 3). The most frequent tips that participants accessed to help with their goals were how to wind down before bed and how to help with being a night owl (Table 4).

**Table 3:** Frequency of goal use by participants (N=83).

Goal			Participants, n (%)
**Reduce schedule variability (jet lag)**			34 (41)
	Reduce to <1 hour	11 (13)	
	Reduce to <2 hours	18 (22)	
	Reduce to <3 hours	5 (6)	
**Limit time in bed**			11 (13)
	Spend 8.5-10.5 hours on weekdays	4 (5)	
	Spend 8.5-10.5 hours on both weekend days and weekdays	7 (8)	
Manage sleepiness			4 (5)
**Target lingering in bed^a^**			16 (19)
	Limit lingering to 30 minutes after alarm	11 (13)	
	Set multiple alarms around room	1 (1)	
	Ask others to help them get up	0 (0)	
	Increase light exposure for first 30-60 minutes	3 (4)	
	Get moving for the first 30 minutes	5 (6)	
	Take a shower within first 30 minutes	4 (5)	
	Remind self not to rely on initial feelings upon waking	5 (6)	
**Reduce naps**			17 (20)
	No naps	12 (14)	
	Decrease number of naps	1 (1)	
	Decrease length of naps	4 (5)	

^a^Participants were free to select as many strategies as they desired from a drop-down list.

**Table 4:** Frequency of tip use by participants (N=83).

Tip access	Participant, n (%)
Completed night owl quiz	17 (20)
Completed difficulty winding down quiz	24 (29)
Completed difficulty getting up quiz	13 (16)
Completed sleep drunkenness quiz	0 (0)
Completed trouble staying awake quiz	5 (6)
Completed fatigue quiz	13 (16)

**Figure 3:**
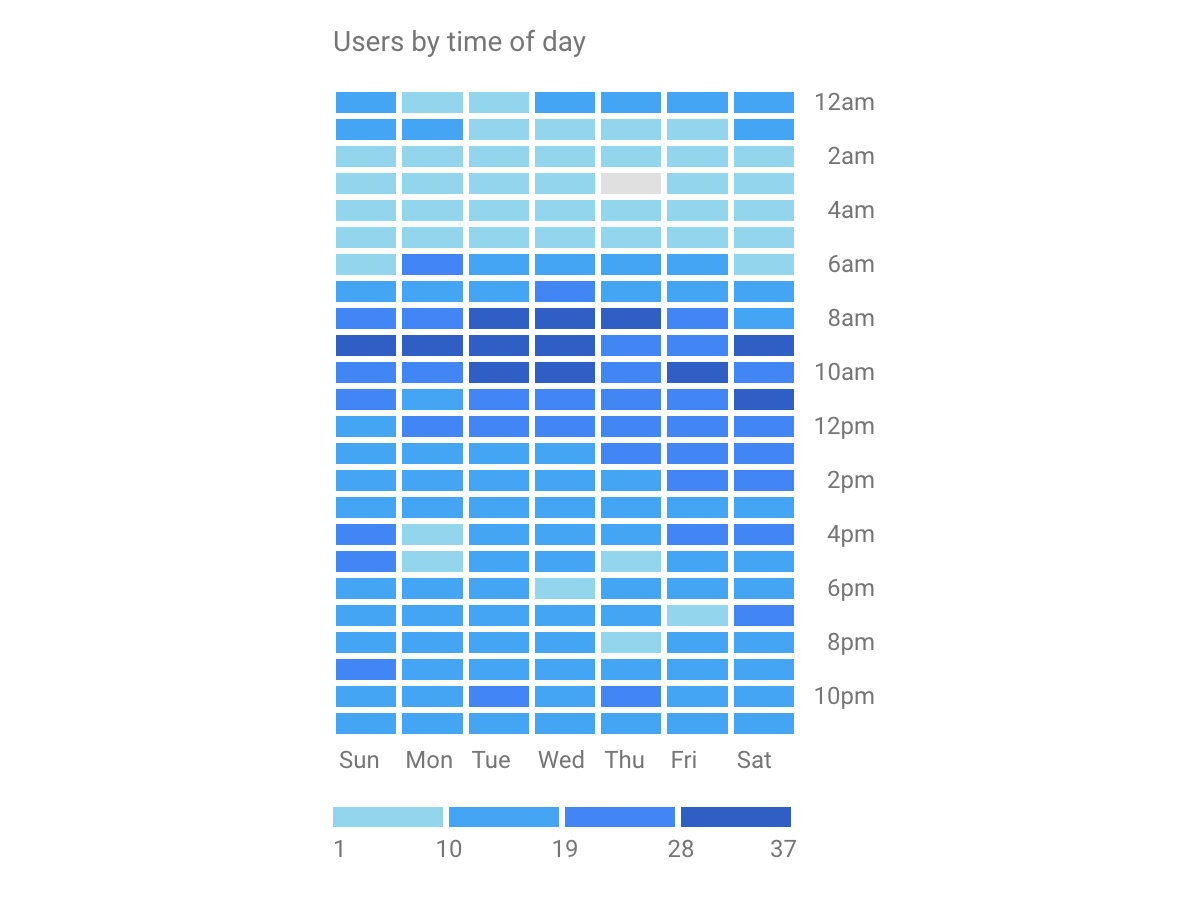
Users of Delivering Online Zzz’s with Empirical Support by time of day.

Google Analytics indicated that 2659 sessions (ie, instances of app use) took place. The average session duration was 3:02 minutes. During the study period, there were 27,263 page views. On average, 10.91 pages were viewed per session (including multiple visits to the same page). When grouped according to content categories, pages related to the sleep diary accounted for most of the 27,263 page views (18,018/27,263, 66.09%), indicating that it was the most used component of the app, followed by Progress (4098/27,263, 15.03%), Dashboard (1925/27,263, 7.06%), Tips (1061/27,263, 3.89%), Goals (810/27,263, 2.97%), Log-In (777/27,263, 2.85%), onboarding screens (431/27,263, 1.58%), and visits to their user profile (142/27,263, 0.52%). The average time on a page was 18.15 seconds across all pages, ranging from 65.51 seconds for progress pages to 6.68 seconds for onboarding pages. DOZE was most frequently accessed using a mobile device (2,705/2659 sessions, 78.04%), followed by a desktop computer (546/2,659 sessions, 20.53%) or a tablet (38/2,659 sessions, 1.43%). Consistent with the sleep diary and in-app recommendations to complete the sleep diary within 2 hours of waking up, DOZE was most frequently visited between 8 AM and 11 AM (Figure 3), with a modal time of 9 AM.

#### Acceptability, Credibility, and Satisfaction

To assess the acceptability, credibility, and satisfaction of DOZE, we analyzed the mean, mode, and range of responses for each item of the TEQ and the Acceptability Scale (Table 5). Responses to the TEQ were favorable at baseline and end point, with mean and modal values exceeding 4 for most items. There was no statistical change from baseline to end point on items assessing logic (*P*=.54), confidence that it would work (*P*=.48), confidence in recommending the app to a friend (*P*=.74), and how successful they predicted it would be in helping others with their sleep (*P*=.78), suggesting that most perceptions of DOZE as a credible and satisfactory intervention were stable over time. However, there was a statistically significant change in the item assessing willingness (*P*=.008). Responses on the Acceptability Scale indicated agreement or high agreement (mode=4 or 5) that DOZE was easy to use, easy to understand, required a reasonable amount of time, and that users were generally satisfied with the app, whereas responses were mostly neutral (mode=3) for enjoyment using the app and how helpful it was in describing their symptoms and quality of life. Averages for each item on the Acceptability Scale ranged from 3.61 (helpful in describing symptoms) to 4.35 (understandable).

**Table 5:** Participants’ responses on the Treatment Evaluation Questionnaire and Acceptability Scale at baseline and during end point assessments (N=51).

Index			Baseline					End point					*P* value
			Value, mean (SD; range)		Value, mode		Value, mean (SD; range)			Value, mode			
	How logical^a^	4.47 (1.72; 1-7)		5		4.71 (1.63; 1-7)			4		.54		
	Confident in success^b^	4.04 (1.71; 1-7)		4		3.96 (1.48; 1-7)			4		.48		
	Recommend to friend^c^	4.37 (1.58; 1-7)		5		4.53 (1.55; 1-7)			5		.74		
	How willing^d^	5.51 (1.73; 1-7)		7		4.49 (1.59; 1-7)			5		.008		
	How successful for others^e^	4.46 (1.49; 1-7)		4		4.53 (1.41; 1-7)			5		.78		
	Easy to use	N/A^f^		N/A		4.16 (0.90; 2-5)			5		—^g^		
	Understandable	N/A		N/A		4.35 (0.89; 2-5)			5		—		
	Enjoyable	N/A		N/A		3.80 (0.96; 2-5)			3		—		
	Helpful in describing symptoms and quality of life	N/A		N/A		3.61 (1.08; 2-5)			3		—		
	Amount of time acceptable	N/A		N/A		4.33 (0.91; 2-5)			5		—		
	Overall satisfaction	N/A		N/A		3.92 (0.82; 2-5)			4		—		

^a^How logical does the treatment you are receiving seem to you?

^b^How confident are you that this treatment will be successful in eliminating your insomnia?

^c^How confident would you be in recommending this treatment to a friend?

^d^How willing are you (were you) to undergo this treatment?

^e^How successful do you think this treatment is for treating other people with insomnia?

^f^N/A: not applicable; scale not administered at baseline.

^g^Significance testing not available.

### Preliminary Efficacy

#### Sleep Indices

To assess preliminary efficacy, we compared sleep indices at periods 1 and 2 among study finishers (Table 2). The sleep indices were positively skewed and were therefore log-transformed. They were then re-examined to confirm that the assumption of normality was met (all variables were approximately normal) and subsequently analyzed using paired samples *t* tests, with the exclusion of frequency of sleep-interfering substance use, which was not transformed because of the presence of 0 values. Significant improvements of small-to-medium effect sizes were seen for log-transformed morning lingering (*P=*.03), TIB (*P=*.02), TST (*P*<.001), SE (*P=*.001), and TWT (*P=*.01), whereas variability in sleep schedule (ie, bedtime, sleep attempt, wake time, rise time), naps, and frequency of sleep-interfering substance use did not significantly change (Table 2).

#### Self-report Measures

Finally, we compared self-report measures at baseline and end point among study finishers (Table 6). To account for slight deviations from normality among some of the variables, paired *t* tests were conducted using bootstrapping. Mean differences (MDs) were resampled 1000 times, and 95% CIs were generated. Statistically significant improvements were seen on the ISI (MD 3.92, SE 0.61; 95% CI 2.82-5.17), CSM (MD −2.15, SE 0.64; 95% CI −3.41 to −0.96), STICSA State (MD 2.36, SE 1.13; 95% CI 0.16-4.64), STICSA Trait (MD 3.97, SE 0.97; 95% CI 2.03-5.87), CESDR-10 (MD 3.10, SE 0.99; 95% CI 1.20-5.18), and SF-36 energy (MD −6.54, SE 2.48; 95% CI −11.28 to −1.79). Changes from baseline to end point were not significant for the remaining measures of fatigue, sleepiness, or health-related quality of life.

**Table 6:** Comparison of self-reported measures from baseline to end point for study finishers.

Self-report measure			Baseline					End point					*P* value			Cohen *d*
			Score, mean (SD)		Cronbach α		Score, mean (SD)			Cronbach α						
ISI^a^			11.65 (4.35)		.77		8.24 (3.95)			.74		.001			0.90	
FSS^b^			4.55 (1.04)		.85		4.35 (1.04)			.86		.07			0.20	
CSM^c^			30.80 (7.37)		.86		32.47 (7.79)			.88		.006			0.43	
**CASQ^d^ total**			44.14 (9.49)		.83		43.14 (11.09)			.89		.50			0.15	
	Sleepiness	27.42 (7.34)		.81		26.54 (8.49)			.88		.63			0.14		
	Alertness	16.74 (3.80)		.77		16.34 (4.23)			.81		.86			0.12		
CESDR-10^e^			15.62 (8.34)		.89		12.98 (7.89)			.90		.004			0.44	
**STICSA^f^**																
	State	39.69 (11.39)		.90		37.83 (13.32)			.94		.05			0.19		
	Trait	40.80 (11.60)		.91		38.83 (14.25)			.94		.002			0.27		
**SF-36^g^**																
	Physical functioning	84.22 (22.96)		.93		88.43 (20.70)			.95		.09			0.28		
	Role functioning-physical	65.20 (39.71)		.85		70.59 (39.28)			.89		.27			0.14		
	Role functioning-emotional	43.33 (44.29)		.88		42.16 (43.12)			.85		.85			0.03		
	Energy	38.30 (18.16)		.77		43.92 ()19.17			.75		.01			0.38		
	Emotional well-being	54.35 (21.27)		.83		56.63 (23.21)			.90		.39			0.13		
	Social functioning	64.22 (27.62)		.81		67.40 (27.17)			.81		.29			0.14		
	Pain	77.45 (15.33)		.64		76.23 (18.48)			.77		.73			0.08		
	General health	62.27 (23.58)		.84		63.95 (22.89)			.77		.08			0.10		

^a^ISI: Insomnia Severity Index.

^b^FSS: Fatigue Severity Scale.

^c^CSM: Composite Scale of Morningness.

^d^CASQ: Cleveland Adolescent Sleepiness Questionnaire.

^e^CESDR-10: Center for Epidemiologic Studies Depression Scale–Revised 10-item Version for Adolescents.

^f^STICSA: State-Trait Inventory of Cognitive and Somatic Anxiety.

^g^SF-36: RAND 36-item Short Form Health Survey 1.0.

## Discussion

### Principal Findings

We tested the feasibility, acceptability, and credibility of a self-management behavioral sleep web-based app designed by and for AYAs (aged 15-24 years). The results suggest that AYAs rated the intervention as logical, had confidence that the self-management approach would work, and had confidence in recommending DOZE to a friend. Willingness significantly decreased from baseline to end point, perhaps reflecting different stages of readiness to implement the strategies provided, environmental limitations that can influence their ability to implement strategies (eg, early school rise times and high academic demands), and intrinsic values that are a barrier to engaging in the intervention (eg, valuing sleeping in on weekends over maintaining a regular schedule). However, it is important to note that the TEQ was completed before participants received any detailed information about the intervention (they were only aware that it was a new sleep app for teens and young adults), representing only naïve expectations of DOZE, whereas at end point they were familiar with the intervention components. DOZE was rated as highly acceptable across indices of ease of use, understandability, time commitment, and satisfaction. Perhaps not surprisingly, ratings about DOZE being enjoyable or succinct in describing their particular symptoms or quality of life issues were acceptable but slightly more modest than ease of use, low time commitment, and overall satisfaction. In support of feasibility, participants engaged with DOZE often (indicated by the number of sessions), most frequently visiting pages pertaining to the sleep diary or their progress and did so using various electronic devices (ie, mobile phones, computers, and tablets). Moreover, although this was not a clinical sample, prospective sleep diary insomnia indices significantly improved from period 1 to period 2 (eg, time spent awake and SE), and the amount of sleep they obtained increased too. AYA users reported significant improvements from baseline to end point in insomnia symptom severity, chronotype, depression, anxiety, and energy. Despite not recruiting a clinical sample, participants in this study overwhelmingly were not treatment-seekers; however, they evidenced very poor health-related quality of life indices and were above clinical cutoffs for insomnia and fatigue, consistent with what we see in the literature [71].

These results are supportive of self-management. AYAs logged on frequently and completed the diary at the instructed time (ie, within 2 hours of waking up in the morning). Moreover, they worked hard at (1) tracking their sleep (ie, tracking for approximately 10 days in each 14-day period) and (2) setting goals that treatment providers would set (eg, limiting schedule variability and naps and decreasing lingering in bed). They accessed tips to help with their goals, particularly how to wind down before bed, how to help with being a night owl, how to get up regularly in the morning, how to help with fatigue, and how to stay out of bed early in the evening. Thus, we can trust AYAs to set their own goals and make behavioral changes to achieve these goals when they are provided with the tools and education to do so autonomously. Trusting patients to make good choices may not come naturally for providers, but the literature suggests that many patients want autonomy in their health care choices [72] and produce good results when given tools that support their autonomy. As a research group, this was an instructive part of the process, and it was driven by both the literature and collaboration with our AYA advisors and stakeholders throughout the co-design process. Perhaps these findings can inform the tailoring of in-person strategies for AYAs to achieve better outcomes.

Statistically significant differences in sleep indices from period 1 to period 2 seemed unlikely, as we recruited a sample that was not selected for clinical levels of sleep disturbance, and we assumed there would be too little variability in these indices to detect significant effects. Furthermore, detecting a change in variables, such as SOL, naps, or TIB, in a nonclinical or subclinical sample in which some have opposing problems (eg, hypersomnia vs insomnia) is also surprising. For example, those with hypersomnia or sleepiness have SOL values that are too low, and those with insomnia have elevated SOL values; use of DOZE could result in changes in opposite directions and, when averaged across participants, appear to have affected no change at all. These problems can also occur in the same AYA during the same week, for example, sleepiness on some nights and insomnia on others. Similarly, some AYAs are told to eliminate naps, and others are told to increase them, resulting in low variability. Therefore, it is encouraging that insomnia indices, such as lingering in bed in the morning, TWT, TST, TIB, and SE, improved. Although AYAs set goals to decrease the variability in their sleep schedules, the improvement did not reach statistical significance. Perhaps this is why they accessed tips about how to get up in the morning (ie, as it is difficult for them to rise at a regular time), and our plan is to expand tips and resources for AYA users on the DOZE website.

An important implication of this research is that this web-based app may be an important tool to increase access to evidence-based interventions. Some AYAs prefer to use technology for health-related behavior change [34,73,74], and smartphone apps appear to have a high uptake potential; 78.04% used a mobile device for DOZE (2,705/2659 sessions), whereas only 20% used a desktop computer (546/2,659 sessions, 20.5%) and 1.4% used a tablet (38/2,659 sessions). Incorporating AYAs in the co-design process is also a notable strength of this web-based app and may promote continued uptake. That said, future research will need to determine whether there would be health disparities based on access to technology, Wi-Fi, and geographical location. Clinicians report favorable attitudes toward CBT-I apps [75], suggesting the possibility of widespread access to evidence-based sleep treatments. Given the limited number of expert providers in this unique group (ie, not pediatric but not fully adult either) and the need for sleep solutions at the level of primary care and community mental health clinics, this web-based app holds the promise of increasing access to tailored, age-specific care. We intend to conduct a health economic study on the impact of DOZE in the future.

### Limitations

There was a 39% (32/83 participants) dropout rate in the self-management intervention portion of the study, and data were not collected on reasons for discontinuation, although this would certainly be valuable. Although we would prefer a lower rate, the rate was lower than that in other sleep app studies in the literature. For example, digital interventions for insomnia have reported rates >50% [76]. Across adolescent mobile health apps more generally, in a study using an internet-delivered, self-help CBT intervention for chronic pain among adolescents (aged 12-17 years), 52% of participants dropped out of the treatment portion of the study [77]. The dropout rate in our study is perhaps not surprising as they were not treatment-seekers, so if their sleep was not a problem, they might have little motivation except for the CAD $50 (US $39.17) compensation for participation. The brief intervention period in this study may also have contributed to lower rates of dropout compared with other studies. In addition, participants in this study were emailed by the research coordinator if they did not complete sleep diaries for several consecutive days; as such, adherence may be lower when disseminated in the real world. A future trial could evaluate whether dropout rates are lower in a clinical sample. Moreover, much of the dropout in this study occurred during the baseline questionnaire period, suggesting that the self-report battery was too burdensome. These questionnaires are not part of the app, so this barrier to participation is not present with regular, real-world app use, and future studies can verify if the app dropout rate is actually lower in real-world app use.

Although appropriate for feasibility trials, the use of an open trial suggests caution when interpreting positive outcomes. Without a control group, we could not ascertain whether the results were specific to the app or the increased sleep attention. In addition, we recruited participants using various strategies (eg, through a university, the community, and mental health providers, which are the intended disseminators of DOZE). Although this could be seen as a strength, given that our sample likely reflects those who will use DOZE in the real world, it may also be a limitation as these could be considered 3 distinct samples. Without knowing more about these samples, we could not test for possible differences. In the same vein, although our intended users are all AYAs, we are cautious about stating that this would be effective in patients, as this was a community sample; although most participants experienced symptoms that were above clinical cutoffs, they were neither clinically assessed nor were they treatment-seekers. We intend to study the properties of the app in those who are treatment-seeking. Our treatment provider stakeholders suggested that they would be most interested in using the diary as an adjunct to their treatment, much like sleep diary apps in adults [78]. DOZE could be used as a tracking and posttherapy relapse prevention tool, with providers prescribing behavioral change rather than the AYA deciding their own treatment course. Given the strong preferences for self-management in this group, it would be interesting to test outcomes with providers versus self-management.

Our findings may also have been influenced by extraneous factors. First, several items in our measure assessing credibility reference specifically to treatment for insomnia and may not have applied equally to all participants, thereby influencing their responses. Moreover, a small percentage of participants were seeing a health care provider for the purpose of a mental disorder or a sleep disorder, which may have also influenced our results given the bidirectional relationship between sleep and psychopathology. Finally, we did not query medication use and could not rule out the possibility that some participants were using sleep medication, which may have influenced our results; although low levels of treatment-seeking for a sleep disorder suggests that this is unlikely, and participants enrolled in the trial as they were dissatisfied with their sleep despite current treatments, it nevertheless remains a possibility among a small minority of our participants.

### Conclusions

In conclusion, given that there were statistically and clinically significant improvements in a nonclinical sample, it is promising for the tool to be used as designed: as a transdiagnostic tool for young adults to engage in sleep self-management digitally. Even those without sleep disorders were interested and effective in making health-related changes, which can translate into improved sleep habits and potential prevention, as well as potentially improving mental wellness in AYAs. Web-based interventions are an acceptable and effective method of facilitating access to evidence-based care among *invisible groups*, with the ultimate effect of reducing the public health burden.

## Appendix

Multimedia Appendix 1 Delivering Online Zzz’s with Empirical Support co-design process.

## Abbreviations

AYA: adolescent and young adult
CASQ: Cleveland Adolescent Sleepiness Questionnaire
CBT-I: cognitive behavioral therapy for insomnia
CESDR-10: Center for Epidemiologic Studies Depression Scale–Revised 10-item Version for Adolescents
CSM: Composite Scale of Morningness
DOZE: Delivering Online Zzz’s with Empirical Support
FSS: Fatigue Severity Scale
ISI: Insomnia Severity Index
MD: mean difference
SE: sleep efficiency
SF-36: RAND 36-item Short Form Health Survey 1.0
SOL: sleep onset latency
STICSA: State-Trait Inventory of Cognitive and Somatic Anxiety
TEQ: Treatment Evaluation Questionnaire
TIB: time in bed
TST: total sleep time
TWT: total wake time
