# Supplementary material for: A Transdiagnostic Self-management Web-Based App for Sleep Disturbance in Adolescents and Young Adults: Feasibility and Acceptability Study
Source: JMIR Form Res. 2021 Nov 1;5(11):e25392. doi: 10.2196/25392 (PMC8694239; doi:10.2196/25392)
Supplement: Multimedia Appendix 1 [file formative_v5i11e25392_app1.doc]

Appendix 1: DOZE Co-Design Process

The original design of DOZE was created by an expert in behavioural sleep medicine (CEC) at Ryerson University and funded by the Canadian Institutes of Health Research and included a sleep diary that provided summaries of sleep indices. Two pilot testing sessions with AYAs were conducted to collect feedback on the app with respect to user experience, usability and potential barriers. The first session consisted of a single work session in which AYAs interacted with the app (*N*=11, 10 females, aged 18 to 26) and the second consisted of a week of using the app (*N*=9, 6 females, aged 15-17 years). Next, a group of experts and healthcare provider stakeholders in the community provided feedback on what they think are the treatment needs of the AYAs with which they work, as well as what would be important for uptake of the app. An industry partner (PIVOT Design) recruited AYAs between the ages of 15 and 24 years from the community to provide their opinions on health-related apps in order to design a free sleep app for AYAs. During this visit, participants answered questions about their sleep and their experience with tracking their health and reported their preferences and thoughts about apps designed for use on smartphones/tablets. Additionally, participants completed a survey reporting which features they thought were most important for a sleep app for AYAs (participants selected from a list of options, rank ordered their selections, and also provided their input using an open-text box) and which AYA sleep treatment needs were most important (participants were given a list of 20 options and were asked to rank their selections). The initial consultations with AYA stakeholders revealed that in addition to improving initial graphics and usability, AYAs wanted an app that provides feedback on their personal sleep habits (over and above feedback on their sleep), opportunities for goal setting, and tips on how to make changes to their sleep.

Following this initial phase, a user-informed redesign of the app was conducted with the following guiding principles: the app was to be 1) evidence-based, and 2) client/user-driven. Six user assessments were conducted over the course of a year on iterative prototype versions of the app; each assessment included two groups (*n*=5 each) of AYAs. The first two assessments included participants between the ages of 15 and 24 years who reported using apps on their smartphones. Participants completed questionnaires assessing their current use of their phone and apps, and they were then asked to complete tasks on (i.e., navigate) the app prototype and provide their impressions during the tasks. The top three description themes of the app that emerged were that it was: 1) informative, 2) useful, and 3) simple, and AYA users most liked 1) the quizzes, 2) the ease of navigation, and 3) the goal setting. Following these two assessments, the top changes suggested by the AYAs were incorporated and this testing procedure was repeated on two more occasions (final overall *N*=30). The prototype was then reviewed by the healthcare provider stakeholders and their feedback was incorporated, resulting in the final version of DOZE.
